# Mapping of the FGF14:Nav1.6 complex interface reveals FLPK as a functionally active peptide modulating excitability

**DOI:** 10.14814/phy2.14505

**Published:** 2020-07-15

**Authors:** Aditya K. Singh, Paul A. Wadsworth, Cynthia M. Tapia, Giuseppe Aceto, Syed R. Ali, Haiying Chen, Marcello D'Ascenzo, Jia Zhou, Fernanda Laezza

**Affiliations:** ^1^ Department of Pharmacology & Toxicology Università Cattolica del Sacro Cuore Rome Italy; ^2^ M.D.‐Ph.D. Combined Degree Program Università Cattolica del Sacro Cuore Rome Italy; ^3^ Biochemistry and Molecular Biology Graduate Program Università Cattolica del Sacro Cuore Rome Italy; ^4^ NIEHS Environmental Toxicology Training Program Università Cattolica del Sacro Cuore Rome Italy; ^5^ Institute of Human Physiology Università Cattolica del Sacro Cuore Rome Italy; ^6^ Department of Neuroscience Università Cattolica del Sacro Cuore Rome Italy; ^7^ Fondazione Policlinico Universitario A. Gemelli IRCCS Rome Italy; ^8^ Center for Addiction Research University of Texas Medical Branch Galveston TX USA; ^9^ Center for Neurodegenerative Diseases University of Texas Medical Branch Galveston TX USA

**Keywords:** accessory protein, excitability, inactivation, sodium channels

## Abstract

The voltage‐gated sodium (Nav) channel complex is comprised of pore‐forming α subunits (Nav1.1–1.9) and accessory regulatory proteins such as the intracellular fibroblast growth factor 14 (FGF14). The cytosolic Nav1.6 C‐terminal tail binds directly to FGF14 and this interaction modifies Nav1.6‐mediated currents with effects on intrinsic excitability in the brain. Previous studies have identified the FGF14^V160^ residue within the FGF14 core domain as a hotspot for the FGF14:Nav1.6 complex formation. Here, we used three short amino acid peptides around FGF14^V160^ to probe for the FGF14 interaction with the Nav1.6 C‐terminal tail and to evaluate the activity of the peptide on Nav1.6‐mediated currents. In silico docking predicts FLPK to bind to FGF14^V160^ with the expectation of interfering with the FGF14:Nav1.6 complex formation, a phenotype that was confirmed by the split‐luciferase assay (LCA) and surface plasmon resonance (SPR), respectively. Whole‐cell patch‐clamp electrophysiology studies demonstrate that FLPK is able to prevent previously reported FGF14‐dependent phenotypes of Nav1.6 currents, but that its activity requires the FGF14 N‐terminal tail, a domain that has been shown to contribute to Nav1.6 inactivation independently from the FGF14 core domain. In medium spiny neurons in the nucleus accumbens, where both FGF14 and Nav1.6 are abundantly expressed, FLPK significantly increased firing frequency by a mechanism consistent with the ability of the tetrapeptide to interfere with Nav1.6 inactivation and potentiate persistent Na^+^ currents. Taken together, these results indicate that FLPK might serve as a probe for characterizing molecular determinants of neuronal excitability and a peptide scaffold to develop allosteric modulators of Nav channels.

## Graphical abstract

FLPK binds to the V160 residue in the FGF14 core domain at the protein:protein interaction interface of the FGF14:Nav1.6 complex. FLPK interferes with FGF14:Nav1.6 complex formation and prevents FGF14‐dependent modulation of Nav1.6 currents. FLPK binds to the FGF14 core domain, but its functional activity requires the FGF14 N‐terminal tail, a molecular determinant of Nav1.6 inactivation. In medium spiny neurons, FLPK significantly increases firing frequency suggesting the ability of the tetrapeptide to modulate Nav1.6 availability during repetitive firing.

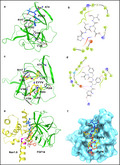

## 1 INTRODUCTION

Voltage‐gated sodium (Nav) channels are transmembrane proteins that mediate the influx of sodium ions in excitable cells, serving as molecular determinants of the action potential. Native Nav channels are composed of a pore‐forming α‐subunit (220–260 kDa), auxiliary β‐subunits (32–36 kDa) (Catterall, 2000; Yu, 2005; Yu & Catterall, 2003) and a matrix of regulatory proteins, such as the intracellular fibroblast growth factors (iFGFs; FGF11‐FGF14; Ali, Shavkunov, Panova, Stoilova‐McPhie, & Laezza, 2014; Goetz et al., 2009; Liu, Dib‐Hajj, & Waxman, 2001, 2003; Rush et al., 2006; Wittmack, 2004). To date, nine isoforms of Nav channels (Nav1.1–1.9) have been functionally characterized and a tenth (Nax) has been identified (Catterall, 2013, 2014; Catterall, Goldin, & Waxman, 2005; Chahine, Chatelier, Babich, & Krupp, 2008; Cusdin, Clare, & Jackson, 2008; Denac, Mevissen, & Scholtysik, 2000; Goldin et al., 2000; Leterrier, Brachet, Fache, & Dargent, 2010; Marban, Yamagishi, & Tomaselli, 1998; Savio‐Galimberti, Gollob, & Darbar, 2012; Yu & Catterall, 2003). These isoforms exhibit differential distribution (Felts, Yokoyama, Dib‐Hajj, Black, & Waxman, 1997) and unique electrophysiological properties (Catterall et al., 2005) that account for cell type specific signatures in sodium currents, such as Nav1.6‐mediated resurgent and persistent currents, and related variations in intrinsic firing pattern (Catterall et al., 2005; England & De Groot, 2009; Lewis & Raman, 2011).

Previous studies have provided evidence for FGF14 as an accessory subunit and a functional regulator of the Nav1.6 channel (Ali, Singh, & Laezza, 2016; Goetz et al., 2009; Laezza et al., 2009; Lou et al., 2005; Rush et al., 2006; White, Brown, Bozza, & Raman, 2019; Wittmack, 2004). Through direct protein:protein interactions (PPI) with the intracellular C‐terminal tail of Nav1.6, the FGF14‐1b isoform controls Nav1.6‐mediated transient currents, kinetics of fast inactivation and voltage‐dependence of activation and steady‐state inactivation of the channel (Ali et al., 2016, 2018). Most of these phenotypes were found to be abolished by a single FGF14^V160A^ mutation with complete inhibition of FGF14 regulation of Nav1.6 currents requiring Ala silencing at both FGF14^V160^ and FGF14^Y158^ (Ali et al., 2016). Corroborated by homology modeling predictions and structural studies of other highly homologous iFGFs (Goetz et al., 2009), these studies led to the conclusion that FGF14^V160^ and FGF14^Y158^ are part of the PPI interface that confers structure–function specificity to the FGF14:Nav1.6 complex.

All macromolecular complexes acquire functional specificity through PPI interfaces (Rattray & Foster, 2018; Rosell & Fernández‐recio, 2018). Consequently, considerable efforts have been devoted to developing probes targeting these key structural determinants (Andrei et al., 2017). Studies have demonstrated that PPI‐based probes are useful tools to study protein complexes in vivo, as well as their translational potential by serving as chemical scaffolds for drug development (Athanasios, Charalampos, Vasileios, & Ashraf, 2017). However, while there has been some success in generating such probes targeting cytoplasmic enzymes (Miller et al., 2017; Petit et al., 2018; Stevers et al., 2018), less progress has been made toward developing PPI‐based modulators of ion channels. Notable exceptions include those targeting the STIM‐Orai‐activating region (SOAR) of the ORAI channel (Zhou et al., 2015, 2018), the sigma‐1 receptor and Kv1.2 channel interacting domain (Kourrich et al., 2013), and the CRMP2's SUMO motif of the Cav and Nav channels (François‐moutal et al., 2018; François‐Moutal et al., 2018).

Therefore, here we aimed to develop probes targeting the interaction between FGF14 and Nav1.6, a PPI complex that controls intrinsic excitability of medium spiny neurons (MSNs) in the nucleus accumbens (NAc) (Ali et al., 2018) and has been linked to numerous neuropsychiatric disorders (Di Re, Wadsworth, & Laezza, 2017); we pursued three peptides (FLPK, PLEV and EYYV), previously designed based on mapping of the FGF14:FGF14 dimer complex interface (Ali et al., 2014), as potential new tools to interrogate the Nav1.6 channel function in heterologous cells and in the native system. Using a combination of in silico docking, in‐cell split‐luciferase complementation assays (LCA), and surface plasmon resonance (SPR), FLPK was revealed as an inhibitor of the FGF14:Nav1.6 complex, with a mechanism dependent on FGF14^V160^. Whole‐cell patch‐clamp electrophysiology subsequently demonstrated that FLPK inhibits FGF14‐dependent phenotypes of Nav1.6‐mediated currents through a mechanism that requires the FGF14‐1b N‐terminal tail. In medium MSNs of the NAc, FLPK increases firing frequency and potentiates persistent Na^+^ currents suggesting interference of the tetrapeptide with FGF14 N‐terminal tail‐dependent modulation of Nav1.6 inactivation (Pan & Cummins, 2020; White et al., 2019). These new studies identify the FLPK tetrapeptide as a useful tool to probe Nav1.6 channel function and a scaffold for future drug development towards treatment of a wide range of channelopathies associated with Nav channels (Alshammari et al., 2016; Chahine et al., 2008; Di Re et al., 2017; Eijkelkamp et al., 2012; Hsu et al., 2017).

## 2 METHODS

### 2.1 Materials

D‐luciferin was purchased from Gold Biotechnology (St. Louis, MO), prepared as a 30 mg/ml stock solution in phosphate‐buffered saline (PBS), and stored at −20°C. Peptides were synthesized from Zhejiang Ontores Biotechnologies Co. (Yuhang District, Hangzhou, Zhejiang, China). Peptides were dissolved in either 100% Dimethyl sulfoxide (DMSO) or HBS‐P^+^ buffer (100 mM hydroxyethylpiperazine ethane sulfonic acid (HEPES), 150 mM NaCl, 0.005% (v/v) P20, pH 7.4; GE Healthcare Bio‐Sciences, Pittsburgh, PA) to prepare 100 mM stock solutions and stored at −20°. For slice electrophysiology the following glutamatergic and GABAergic synaptic transmission blockers were used; 2,3‐dihydroxy‐6‐nitro‐7‐sulfamoylbenzo (f) quinoxaline (NBQX) disodium salt at a final concentration of 20 µM (Tocris, MN) of prepared from stock solution (100 mM in H_2_O); (+)‐Bicuculline 20 µM (Sigma‐Aldrich, MO) of stock solution (100 mM in DSMO); and D and L forms of 2‐amino‐5‐phosphonovalerate and 2‐amino‐4‐phosphonobutyrate (DL‐AP5) sodium salt 100 µM (Tocris) of stock solution in (H_2_O 100 mM) were stored at −20° and 4°C respectively.

### 2.2 Plasmids

Plasmids used in this study derived from the following clones: human FGF14‐1b isoform (accession number: NM_175929.2) and human Nav1.6 (accession number: NM_014191.3). The CLuc‐FGF14, CD4‐Nav1.6‐NLuc, constructs and the pcDNA3.1 vector (Invitrogen, Carlsbad, CA) were engineered and characterized as previously described (Goetz et al., 2009; Shavkunov et al., 2012,2013,2015; Ali et al., 2016, 2018; Wadsworth et al., 2019). The plasmid pGL3 expressing full length Firefly (Photinus pyralis) luciferase was a gift from Dr. P. Sarkar (Department of Neurology, UTMB). For protein purification studies, cDNAs encoding FGF14‐1b (accession number NP_787125; aa 64–252) or the C‐terminal tail of Nav1.6 (accession number #NP_001171455; aa 1756–1939) were subcloned into suitable pET bacterial expression vectors (pET28a‐FGF14; pET30a‐Nav1.6) with a 6X His‐tag at the N‐terminal site; these plasmids were a gift of Dr. Moosa Mohammadi (NYU, Langone Medical Center). The mutation coding for FGF14^V160A^ was generated by site‐directed mutagenesis and PCR using FGF14‐1b as a template described previously (Ali et al., 2016). For electrophysiological studies FGF14‐GFP (human) and FGF14‐ΔNT‐GFP (human) were cloned into the GFP plasmid (pQBI‐fC2; Quantum Biotechnology Inc., Montreal, Canada) as previously described.

### 2.3 Homology model‐based FLPK docking to FGF14

The docking study was performed with Schrödinger Small‐Molecule Drug Discovery Suite using the FGF14 chain of a previously described FGF14:Nav1.6 homology model (Ali et al., 2016). The protein structure was prepared with Protein Prepared Wizard. FLPK, EYYV, and PLEV peptide fragments (containing N‐terminal acetylation and C‐terminal amidation) were prepared with LigPrep and the initial lowest energy conformation was calculated. The grid center was chosen on the coordinate of X = 27.4, Y = −14.88, Z = −15.97. Grid box size was set to 50 × 50 × 50 Å and a finer scaling factor of 0.5 was used. Grid generation and docking were both employed with Glide using SP‐Peptide protocol. Docking poses were incorporated into Schrödinger Maestro for a visualization of ligand‐receptor interactions. Overlay analysis was performed with the docked pose of FLPK, PLEV, EYYV and FGF14:Nav1.6 homology model using Schrödinger Maestro.

### 2.4 Cell Culture and Transient Transfections

HEK293 cells were maintained in DMEM and F‐12 (Invitrogen, Carlsbad, CA), supplemented with 0.05% glucose, 0.5 mM pyruvate, 10% fetal bovine serum, 100 units/ml penicillin, and 100 µg/ml streptomycin (Invitrogen), and incubated at 37ºC with 5% CO_2_. HEK293 cells stably expressing the human Nav1.6 channel (hereafter referred to as HEK‐Nav1.6 cells) were maintained similarly except for the addition of 500 μg/ml G418 (Invitrogen) to maintain stable Nav1.6 expression. Cells were transfected at 80%–90% confluence with equal amount (1 μg each) of plasmid pairs using Lipofectamine 2000 (Invitrogen) according to manufacturer's instructions. HEK‐Nav1.6 cells were washed and replated at very low density prior to electrophysiological recordings (Ali et al., 2016, 2018; Scala et al., 2018; Wadsworth et al., 2019).

### 2.5 Split‐luciferase Complementation Assay (LCA)

Twenty‐four hours after transfection, HEK293 cells were replated from the 24‐well plate using a 0.04% Trypsin:EDTA mixture dissolved in PBS. Suspended cells were centrifuged and seeded in white, clear‐bottom 96‐well tissue culture plates (Greiner Bio‐One) in 200 µl of medium. The cells were incubated for 24 hr and then the growth medium was replaced with 100 µl of serum‐free, phenol red–free DMEM/F12 medium (Invitrogen, Carlsbad, CA) containing either 0.5% DMSO alone (vehicle) or peptides (50 µM) dissolved to a final concentration of 0.5% DMSO. The bioluminescence reaction was initiated by dispensing 100 µl of D‐luciferin substrate (1.5 mg/ml dissolved in PBS) using a Synergy^TM^ H4 Multi‐Mode Micro plate Reader (Biotech, Winooski, VT). Luminescence readings were initiated after 3 s of mild plate shaking and performed at 2 min intervals for 20 min with integration times of 0.5 s. Cells were maintained at 37°C throughout the measurements. Detailed methods for LCA can be found in previous studies (Ali et al., 2014, 2016, 2018; Hsu et al., 2015; Shavkunov et al., 2012; Wadsworth et al., 2019).

### 2.6 Protein Expression and Purification

Upon transformation with corresponding cDNA clones, the recombinant proteins FGF14, FGF14^V160A^ and Nav1.6 C‐terminal tail were expressed in *E. coli* BL21 (DE3) pLys (Invitrogen) after induction with 0.1 mM isopropyl thio‐β‐D‐galacto‐pyranoside (IPTG) for 24 hr at 16°C. After induction with IPTG, bacterial cells were harvested and lysed by sonication at 4°C in lysis/binding buffer containing following components (mM): sodium phosphate 10 (prepared from 0.5 M of Na_2_HPO_4_ and NaH_2_PO_4_) + 3‐cholamidopropyl dimethylammonio 1‐propanesulfonate 0.1% pH 7.0 (for FGF14 proteins) + HEPES 25 + NaCl 150 + glycerol 10% (Nav1.6) pH 7.5 and containing 0.1 mM phenyl methyl sulphonyl fluoride (PMSF). The respective proteins were centrifuged at 18,000 g for 30 min at 4°C. For purification of FGF14 and FGF14^V160A^, the supernatant was applied to preequilibrated heparin and the proteins were then eluted with NaCl 0.2–2.0 M in the elution (sodium phosphate 10 mM + NaCl 0.2–2.0 M pH 7.0) buffer. For purification of the Nav1.6 C‐tail, the supernatant was applied first to Ni^2+^ NTA column and eluted with imidazole (200 mM). The Nav1.6 C‐tail was further purified using HiTrap QFF‐sepharose column (GE Healthcare Bio‐Sciences, Pittsburgh, PA) with a buffer containing Tris‐HCl 50 mM and eluted with NaCl (10–500 mM) at pH 7.5. Finally, all concentrated proteins were purified on an AKTA FPLC purifier using Superdex 200 Hiload 16 × 60 columns (GE Healthcare Bio‐Sciences) and equilibrated in Tris‐HCl 50 mM + NaCl 150 mM, pH 7.5 (Ali et al., 2016; Scala et al., 2018).

### 2.7 Surface Plasmon Resonance Spectroscopy

SPR experiments were performed on a Biacore T100 (GE Healthcare Bio‐Sciences, Pittsburgh, PA), and the interaction of FGF14 and Nav1.6 channel toward FLPK, PLEV and EYYV (1–100 µM) were studied at 25°C. To analyze the effects of FLPK, PLEV and EYYV on FGF14^WT^ (RU 16,000), FGF14^V160A^ (RU 18,000) and Nav1.6 C‐tail (RU 12,000), the proteins were immobilized on CM5 sensor chip using Acetate 5.5 with Amine Coupling Kit (Biacore GE Healthcare Bio‐Sciences). No protein was coupled to the control flow channel of the chip. Using a flow rate of 50 µl/min, FLPK, PLEV and EYYV (1–100 µM) diluted in HBS‐P^+^ buffer were injected over the chip for 180 s, followed by injection of HBS‐P+ for 180 s to monitor dissociation, and finally the chip surface was regenerated with NaCl (200 mM). For each peptide injection, nonspecific responses (buffer only) were subtracted from experimental sensograms/traces prior to data analysis. Maximal equilibrium responses were plotted against peptide concentrations and association rate constant (k_on_), dissociation rate constatnt (k_off_) were determined. The equilibrium dissociation constant (K_D_) was calculated from the fitted saturation binding curve using kinetic model 1:1 binding and also using Langmuir model (K_D_ = k_off_/k_on_). The kinetic constants generated from the fitted binding curves were assessed for accuracy based on the distribution of the residuals (even and near zero to baseline). Graphs were plotted in GraphPad Prism 7 Software (La Jolla, CA).

### 2.8 Electrophysiology Experiments in Heterologous Cells

HEK‐Nav1.6 cells transfected with GFP or FGF14‐GFP were plated at low density on glass cover slips for 3–4 hr and subsequently transferred to the recording chamber. Recordings were performed at room temperature (20–22°C) 24 hr posttransfection using a MultiClamp 700B amplifier (Molecular Devices, Sunnyvale, CA). The composition of recording solutions consisted of the following salts; extracellular (mM): 140 NaCl, 3 KCl, 1 MgCl_2_, 1 CaCl_2_, 10 HEPES, 10 glucose, pH 7.3; intracellular (mM): 130 CH_3_O_3_SCs, 1 EGTA, 10 NaCl, 10 HEPES, pH 7.3. Membrane capacitance and series resistance were estimated by the dial settings on the amplifier and compensated for electronically by 70%–75%. Data were acquired at 20 kHz and filtered at 5 kHz prior to digitization and storage. All experimental parameters were controlled by Clampex 9.2 software (Molecular Devices) and interfaced to the electrophysiological equipment using a Digidata 1,200 analog‐digital interface (Molecular Devices). Voltage‐dependent inward currents for HEK‐Nav1.6 cells were evoked by depolarization to test potentials between −100 mV and +60 mV from a holding potential of −70 mV followed by a voltage prestep pulse of −120 mV (Nav1.6). Steady‐state (fast) inactivation of Nav channels was measured with a paired‐pulse protocol. From the holding potential, cells were stepped to varying test potentials between −120 mV (Nav1.6) and +20 mV (prepulse) prior to a test pulse to −20 mV.

Current densities were obtained by dividing Na^+^ current (I_Na_) amplitude by membrane capacitance. Current–voltage relationships were generated by plotting current density as a function of the holding potential. Conductance (G_Na_) was calculated by the following equation:

$$G_{\text{Na}}=I_{\text{Na}}/\left(V_{m}‐E_{\text{rev}}\right)$$

where I_Na_^+^ is the current amplitude at voltage V_m_, and E_rev_ is the Na^+^ reversal potential.

Activation curves were derived by plotting normalized G_Na_ as a function of test potential and fitted using the Boltzmann equation:

$$G_{\text{Na}}/G_{\text{Na,Max}}=1/\left[1+e\left[\left(V_{a}‐E_{m}\right)/k\right]\right]$$

where G_Na,Max_ is the maximum conductance, V_a_ is the membrane potential of half‐maximal activation, V_m_ is the membrane voltage and k is the slope factor.

The membrane potential of half‐maximal activation, E_m_ is the membrane voltage and k is the slope factor. For steady‐state inactivation, normalized current amplitude (I_Na_/I_Na,Max_) at the test potential was plotted as a function of prepulse potential (V_m_) and fitted using the Boltzmann equation:

$$I_{\text{Na}}/I_{\text{Na,Max}}=1/\left[1+e\left[‐\left(V_{h}‐E_{m}\right)/k\right]\right]$$

where V_h_ is the potential of half‐maximal inactivation, E_m_ is the membrane voltage, and k is the slope factor.

### 2.9 Electrophysiology Experiments In Brain Slices

Brain slices from male C57BL6/J mice aged 28–45 days were prepared to contain the nucleus accumbens (NAc). Mice were anesthetized with Isoflurane (Baxter, Deerfield, IL) and brains extracted following decapitation. Brains were sliced with a vibratome (Leica Microsystems, Wetzlar, Germany) to achieve 300 μm coronal slices. Brains sliced in extracellular salts; consisting of the following (mM): Tris‐HCl 72, Tris‐Base18, NaH_2_PO_4_ 1.2, KCl 2.5, HEPES 20, sucrose 20, NaHCO_3_ 25, glucose 25, MgSO_4_ 10, Na‐pyruvate 3, Na‐ascorbate 5 and CaCl_2_ 0.5 (Sigma‐Aldrich, St. Louis, MO); 300–310 mOsm, pH 7.4 and was continuously oxygenated (mixture of 95%/5% O_2_/CO_2_). Slices were then placed in a 32°C recovery chamber with freshly prepared tris‐based aCSF for 15 min before transfer to a 32°C chamber of standard aCSF consisting of the following extracellular salts (mM): NaCl 123.9, KCl 3.1, glucose 10, MgCl_2_ 1, CaCl_2_ 2, NaHCO_3_ 24, and NaH_2_PO_4_ 1.16 (Sigma‐Aldrich, St. Louis, MO); 300–310 mOsm, pH 7.4 and continuously oxygenated (mixture of 95%/5% O_2_/CO_2_). Finally, slices were equilibrated at RT for at least 45 min. DMSO and/or FLPK were added to standard aCSF to achieve a bath concentration of 0.1% DMSO and 50 μM FLPK. Brain slices were incubated in a separate chamber with standard aCSF containing 0.1% DMSO (control) or 50 μM FLPK and continuously oxygenated for 30 min. Somatic recordings from NAc visually identified medium spiny neurons (MSNs) in standard aCSF were carried out at 31°C using borosilicate glass pipettes (resistance of 3–5 MΩ) filled with an internal solution containing (mM): D‐gluconate (potassium salt) 145, MgCl_2_ 2, EGTA 0.1, Na_2_ATP 2, and HEPES 10 (pH 7.2; 290 mOsm). Either an Axopatch 200B or a Multiclamp 700B amplifier was used to perform whole‐cell patch clamp experiments. For the Axopatch 200B, a digidata 1,200 analog–digital interface and pClamp 9 software with filtering at 20 kHz and digitizing at 5 kHz were used for data acquisition and stimulation. For the Multiclamp 700B, a digidata 1,350 analog‐digital interface and pClamp 10.9 with filtering at 20 kHz and digitizing at 5 kHz were used for data acquisition and stimulation. First a giga‐seal was formed and the cell membrane ruptured after which MSNs were held in voltage‐clamp mode for 1 min to measure resting membrane potential. Next, MSNs were held in current clamp mode to assess cell activity. Evoked action potentials with a range of current injections from 10 pA to 220 pA with 800 ms every 10 pA were measured and number of action potentials, instantaneous frequency, and other electrical parameters were determined. Average instantaneous firing frequency (IFF) is defined as the average of all reciprocal interspike intervals (ms) at a particular current step. Cells with a current threshold above 180 pA, a resting membrane potential above −60 mV, and an input resistance outside the range of 70–250 MΩ were excluded. To isolate persistent Na^+^ current (*I*_NaP_) in whole‐cell voltage‐clamp recordings, we used a modified aCSF containing 20 mM tetraethylammonium chloride (TEA) and 0.3 mM CdCl_2_. The voltage‐dependence of *I*_NaP_ was determined using standard protocols (Yue, Remy, Su, Beck, & Yaari, 2005). Membrane capacitances and Rseries were compensated electronically. Series resistances (Rs) before compensation were in the range of 5–20 MΩ and were routinely corrected by 75%–85%. Data obtained from a given cell were rejected if Rs was larger than 20 MΩ or changed by > 20% during the course of the experiment. Membrane capacitance was calculated using the equation: capacitance = membrane time constant/input resistance (Meitzen, Weaver, Brenowitz, & Perkel, 2009). The membrane input resistance was measured by a series of 600 ms hyperpolarizing current steps from −50 to 0 pA, step 10 pA with 1 s interval. The slope of the current‐voltage curve was designated as the membrane input resistance. The membrane time constant was calculated by fitting a single exponential curve to the membrane potential change in response to −200 pA hyperpolarizing pulses. All the electrophysiological recordings were analyzed using Clampfit 9 or Clampfit 10.9 software (Molecular Devices, San Jose, CA) and SigmaPlot 14.0 (Systat Software Inc., San Jose, CA).

### 2.10 Data analysis

Statistics were calculated as mean and standard error of the mean (mean ± *SEM*) using Prism 7 (La Jolla, CA) or Origin 8.6 software (OriginLab Corporation, Northampton, MA), unless otherwise specified. The statistical significance of observed differences among groups was determined by Student's *t* test or for two group comparisons significance was tested with unpaired, one‐way ANOVA with *post hoc* Fisher's LSD, *post hoc* Bonferroni or Dunn's test; *p < .05 was regarded as statistically significant. Dose‐response curves were obtained using GraphPad Prism 7 by fitting the data with a nonlinear regression, as described previously (Wadsworth et al., 2019). Electrophysiological data analysis was performed using Clampfit 9 software (Molecular Devices) and Origin 8.6 (OriginLab Corporation). SPR data analysis was done using Biacore evaluation software (Biacore GE Healthcare Bio‐Sciences) and plotted with Prism 7.

### 2.11 Animals

C57/BL6J animals were purchased from Jackson Laboratory (Bar Harbor, ME) and mice were bred in‐house for work done at Università Cattolica del Sacro Cuore. Mice were housed, *n* ≤ 5 per cage, with food and water ad libitum. Mice were closely monitored for health and overall well‐being daily by veterinary staff and the investigators. Animal maintenance, all surgical procedures and experiments were performed in accordance with the US National Institutes of Health (NIH) guidelines and were approved by the Institutional Animal Care and Use Committee (IACUC) of the University of Texas Medical Branch. For work done at Università Cattolica del Sacro Cuore, all animal procedures were approved by the Ethics Committee of Università Cattolica del Sacro Cuore and complied with the Italian Ministry of Health guidelines and with national laws (Legislative Decree 116/1992) and European Union guidelines on animal research (86/609/EEC).

## 3 RESULTS

### 3.1 In silico docking of FLPK, PLEV and EYYV to the FGF14:Nav1.6 PPI interface

Previous studies have provided evidence for a conserved role of the β9‐ β12 strands of FGF14 in structural interactions with Nav1.6 (Ali et al., 2016, 2018) We evaluated in silico interaction of three previously designed peptides mapped to the β9‐ β12 strands at the dimer interface (Ali et al., 2014) in the context of Nav1.6 complex.

**FIGURE 1:**
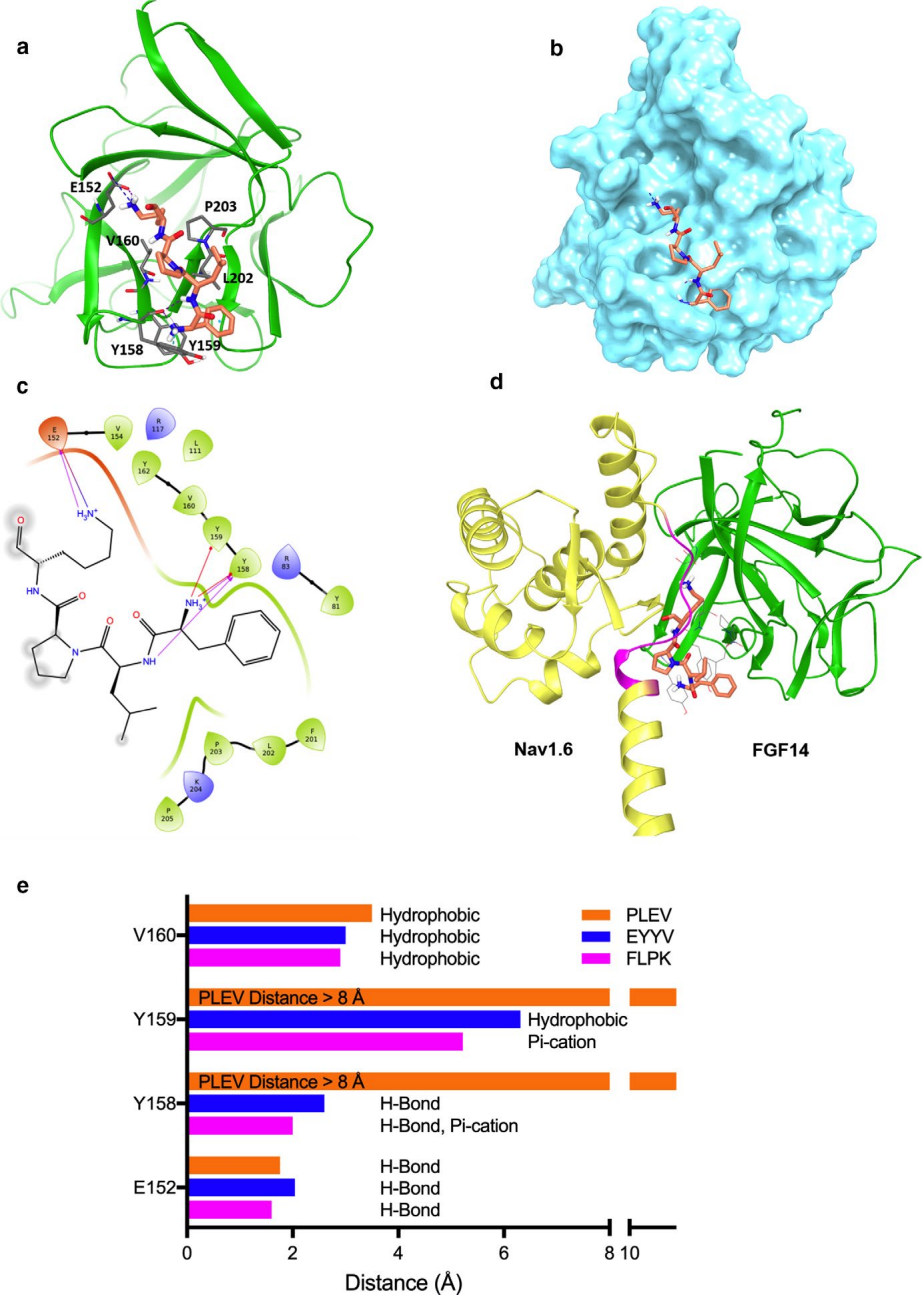
FLPK docking to the FGF14:Nav1.6 C‐terminal tail complex. (a) ribbon presentation of peptide fragment FLPK (orange) docking into the FGF14 chain of the FGF14:Nav1.6 complex homology model. FGF14 is depicted as green ribbons. Key interaction residues are depicted as gray sticks. Hydrogen bonds and π‐cation interactions are depicted as purple and cyan dotted lines, respectively. (b) surface presentation of FLPK docking into FGF14. (c) interaction diagram of the predicted FLPK binding site. Residues shown in the map are within 4 Å cut‐off to FLPK. Hydrogen bonds and π‐cation interactions are depicted as purple and red dotted lines, respectively. (d) overlay of the FLPK docked pose (orange) and the FGF14:Nav1.6 complex homology model. The FGF14 chain and Nav1.6 C‐terminal tail are represented as green and yellow ribbons, respectively. Residues 1883–1892 of the Nav1.6 C‐terminal tail are located at the PPI site and are highlighted in purple. (e) the distance (<8 Å) between each FGF14 hotspot amino acid to the docked FLPK, EYYV, and PLEV peptides was determined using the Schrödinger Small‐Molecule Drug Discovery Suite from a homology model of the FGF14:Nav1.6 complex

**FIGURE 2:**
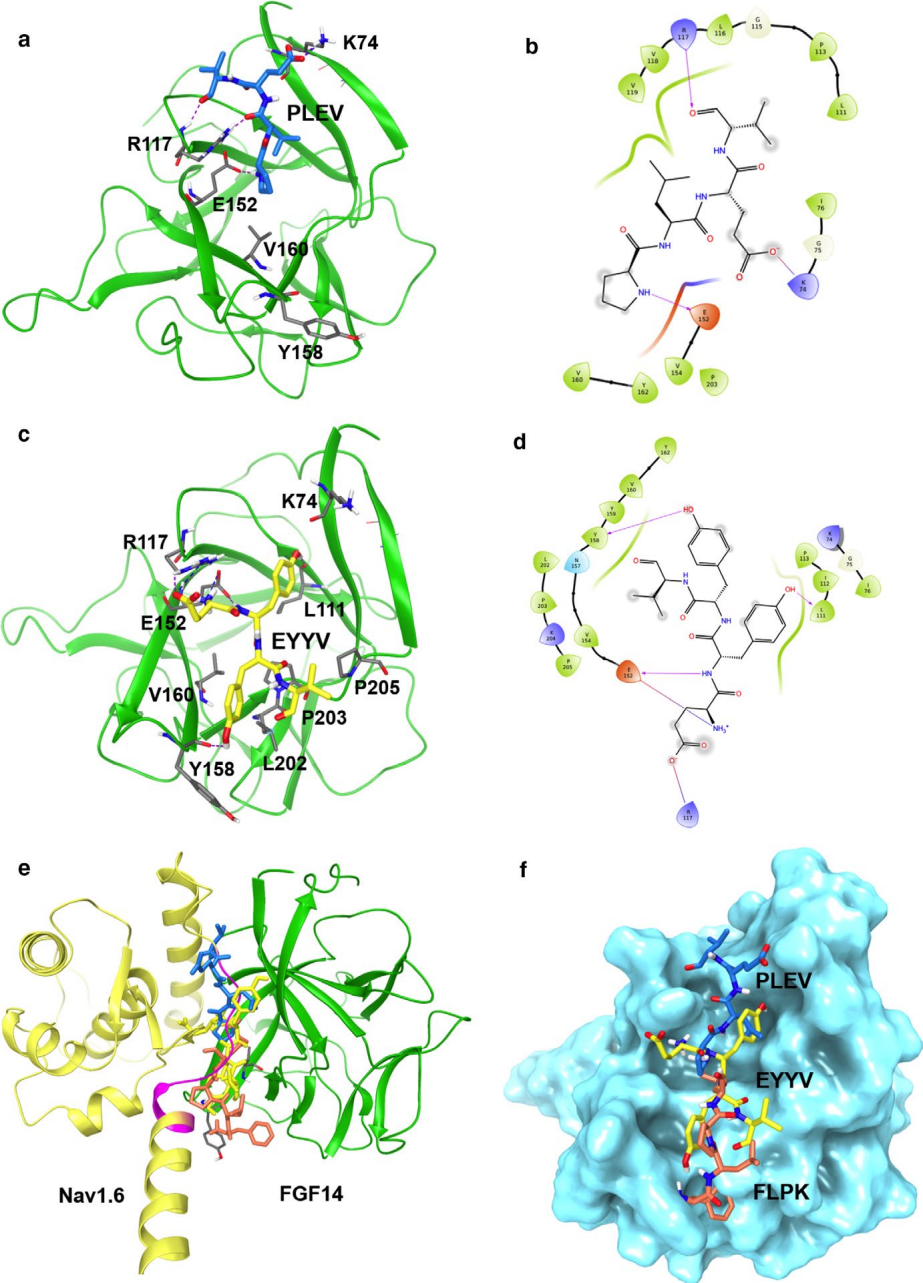
PLEV and EYYV docking to the FGF14:Nav1.6 C‐terminal tail complex. (a) ribbon representation of PLEV (blue) docked into the FGF14 chain of the FGF14:Nav1.6 C‐terminal tail homology model. FGF14 is depicted as green ribbons. Key interaction residues are depicted as gray sticks. H‐bonds are depicted as purple dotted lines, salt bridge is depicted as blue dotted line. (b) interaction diagram of predicted PLEV binding site. Residues shown in map are within 4 Å cut‐off to PLEV. H‐bonds and salt bridges are depicted as purple lines. (c) ribbon presentation of peptide fragment EYYV (yellow) docking into the FGF14 chain of the FGF14.Nav1.6 C‐terminal tail complex homology model. FGF14 is depicted as green ribbons. Key interaction residues are depicted as gray sticks. Hydrogen bonds are depicted as purple dotted lines, salt bridge is depicted as blue dotted line. (d) interaction diagram of predicted EYYV binding site. Residues shown in map are within 4 Å cut‐off to EYYV. H‐bonds and salt bridge are shown in purple. (e) overlay of FLPK (orange), PLEV (blue) and EYYV (yellow) docked poses and FGF14.Nav1.6 C‐terminal tail complex homology model. The FGF14 chain is depicted as green ribbons and Nav1.6 C‐terminal tail is depicted as yellow ribbons. (f) surface presentation of FLPK, PLEV and EYYV docking into FGF14

FLPK, PLEV and EYYV were docked to a homology model of the FGF14:Nav1.6 C‐terminal tail complex derived from previous studies. In order to allow peptides to dock to any portion of FGF14, including residues mediating Nav1.6 C‐terminal tail binding, the Nav1.6 chain was removed from the homology model prior to peptide docking. Subsequently, Nav1.6 was overlaid with the docking results to compare peptide binding with Nav1.6 binding. The docking results demonstrated that FLPK can be well docked into monomeric FGF14 (Figure 1a‐c) within the binding pocket formed by the β9 and β12 strands, specifically by interactions with hotspot residues that are similarly found at the interface of the FGF14:Nav1.6 complex (Ali et al., 2016, 2018). In contrast, while EYYV and PLEV demonstrated minimal binding to the inner pocket, they were predicted to bind more peripheral regions of FGF14 (β5 and N‐terminus) that interact with Nav1.6 (Figure 2 and Table 1).

**TABLE 1:** Assessing homology model‐based hotspot interactions with peptides at the FGF14:Nav1.6 complex

FGF14 Location	FGF14:Nav1.6	FLPK	PLEV	EYYV
β9	N157, Y158, Y159, V160	E152, N157, Y158, Y159, V160 (H‐bonds, π‐cation hydrophobic)	E152 (H‐bond), No interaction with N157, Y158, Y159 and V160	E152 (H‐bond), No interaction with N157, Y158, Y159 and V160
β12	L202, P203, P205, V208	P203 (hydrophobic)	P203 (No interaction)	P203, V208 (hydrophobic)
β5	L116, R117	No	R117 (H‐bond) L116, P113 (hydrophobic)	R117(H‐Bond, salt bridge), P113 (hydrophobic)
N‐terminus	K74, I76	K74 (No interaction) I76 (hydrophobic)	K74 (H‐Bond, Salt Bridge) I76 (No interaction)	K74 (H‐bond), I76 (hydrophobic)

An overview of the interactions between peptides and key residues of the FGF14:Nav1.6 interaction is provided in Table 1. With regard to the β9 strand, all peptides interact with E152 via hydrogen bonds (H‐bonds), but only FLPK interacts with N157 (H‐bond) or the aromatic residues Y158 (H‐bond and π‐cation) and Y159 (π‐cation), as shown in Figure 1e. In addition, FLPK strongly engages in hydrophobic interactions with residues V160 and P203, while EYYV engages in a weaker hydrophobic interaction with P203 (but not V160 despite apparent physical proximity) and PLEV shows no interaction with these hydrophobic residues. Unlike FLPK, the EYYV and PLEV peptides were predicted to dock more strongly with the β5 strand and N‐terminus via H‐bonding with R117 and K74, respectively (Figure 2a‐d).

Figure 1d shows the FLPK docked pose overlaid with the FGF14:Nav1.6 homology model, indicating that FLPK may bind FGF14 by interacting with key residues in the binding pocket similar to Nav1.6. Comparatively, EYYV and PLEV were predicted to interact with FGF14 at regions of the FGF14:Nav1.6 interaction interface that are more peripheral (Figure 2e,f), but nonetheless have been shown to play a role in mediating the protein–protein interactions, at least at the FGF14:FGF14 dimer interface. Based on these results, all three peptides were moved forward for initial screening against the complex in cells.

### 3.2 In‐cell evaluation of FLPK, PLEV and EYYV using LCA

**FIGURE 3:**
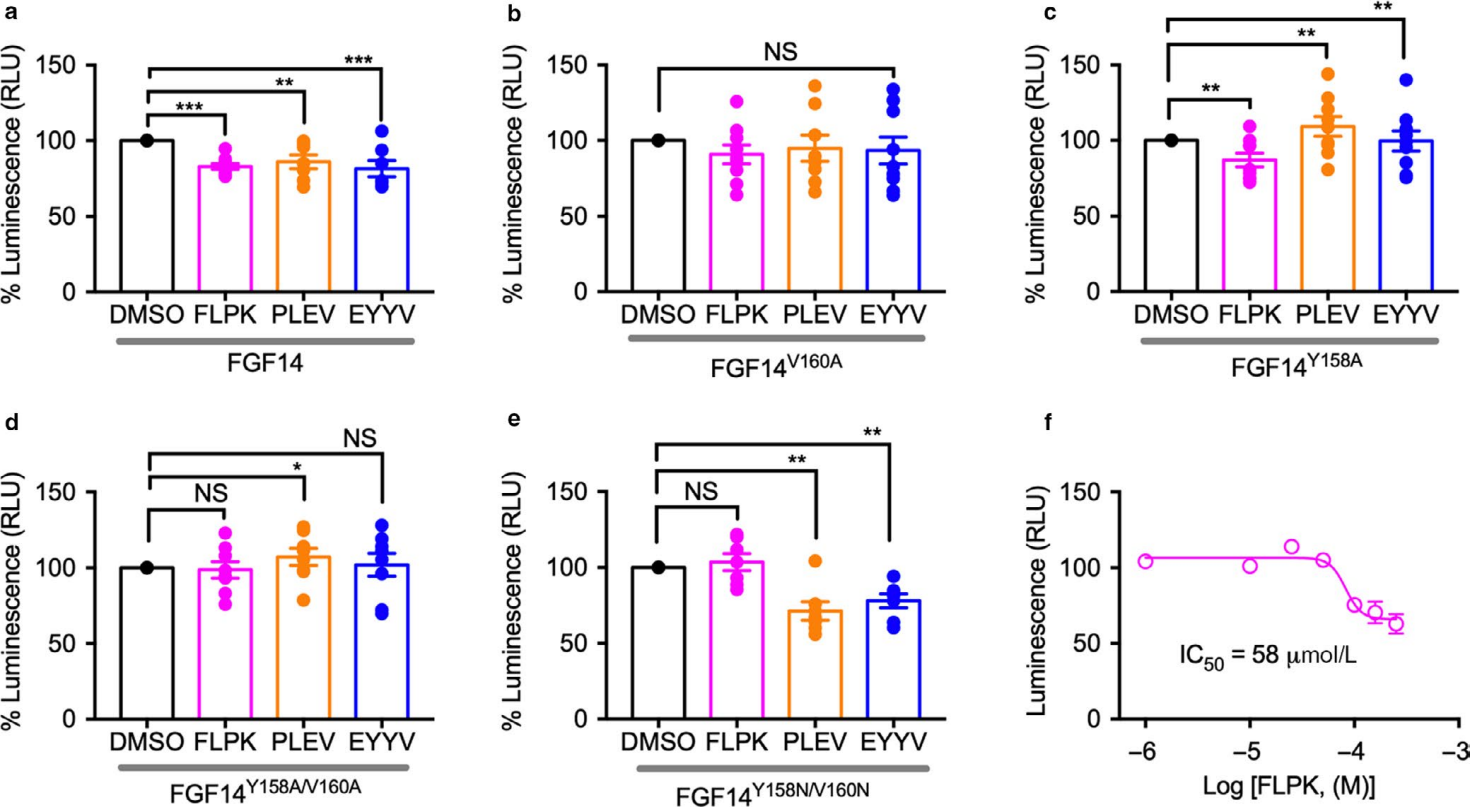
Split‐luciferase complementation assay reveals FLPK as inhibitor of the FGF14:Nav1.6 complex. HEK293 cells were transiently transfected with CD4‐Nav1.6‐NLuc and CLuc‐FGF14 (a,f), CLuc‐FGF14^V160A^ (b), or CLuc‐FGF14^Y158A^ (c), CLuc‐FGF14^Y158A/V160A^ (d), or CLuc‐FGF14^Y158N/V160N^ (e) and treated with peptides (FLPK, PLEV, or EYYV) or 0.5% DMSO alone (vehicle) in 96‐well plates. (a‐e), bar graphs representing percent maximal luminescence response (normalized to DMSO controls) from transfected HEK293 cells treated with peptides (50 µM) or 0.5% DMSO alone. (f) dose‐response for FLPK (1, 10, 25, 50, 75, 100, 150, and 250 µM) against CLuc‐FGF14:CD4‐Nav1.6‐NLuc. The data were analyzed using one‐way ANOVA with *post hoc* Dunnett's analysis (*n* = 6–10 independent experiments; *n* = 4 replicates). Data are mean ± *SEM*. SEMs are shown as error bars in the figures. *p < .05, **p < .01, ***p < .001; NS = nonsignificant. Student's *t* test

To test the hypothesis that FLPK, PLEV and/or EYYV in cells could act as FGF14 inhibitors of the FGF14:Nav1.6 complex assembly, the complex was reconstituted using LCA (Ali et al., 2014, 2016; Hsu et al., 2015, 2017; Shavkunov et al., 2012,2013,2015; Wadsworth et al., 2019). HEK293 cells transfected with constructs expressing CLuc‐FGF14 and CD4‐Nav1.6 C‐tail‐NLuc in which luminescence is detected upon addition of the D‐luciferin substrate as a read‐out of protein binding (Figure 3). In‐cell binding of the reconstituted FGF14:Nav1.6 complex in the presence of vehicle (0.1% DMSO) is represented as summary bar graphs of percent maximal luminescence at 10–15 min of reaction time (Figure 3). Transfected cells were treated with either FLPK, PLEV or EYYV (50 µM, 12 hr) and the resulting luminescence normalized to DMSO vehicle control pair (Figure 3). One‐way ANOVA with *post hoc* Dunn's analysis over a large data set (Figure 3a; *n* = 6–10 independent experiments; *n* = 4 repetitions) revealed a significant reduction of the FGF14:Nav1.6 complex formation in the presence of FLPK, EYYV (**p < .01, One‐way ANOVA with *post hoc* Dunn's analysis) or PLEV (***p < .001, One‐way ANOVA with *post hoc* Dunn's analysis) compared to control vehicle (Figure 3a). Alanine mutations at V160 alone (FGF14^V160A^) or in combination with Y158 (FGF14^Y158A/V160A^) prevented the effect of FLPK and EYYV (Figure 3b and c) while the double FGF14^Y158A/V160A^ reversed the effect of PLEV causing a slight, but statistically significant increase in the FGF14^Y158A/V160A^:Nav1.6 assembly (Figure 3d). Additionally, single Y158A mutation or double FGF14^Y158N/V160N^ mutations, which were used in an effort to separate the electrostatic from the structural component brought by the two residues (Ali et al., 2016; Goetz et al., 2009), increased the inhibition caused by PLEV and EYYV, but abolished the effect of FLPK (Figure 3c,e). On the basis of these results, we conducted a more thorough characterization of FLPK, by constructing a 7‐point dose‐response curve. Out of the resulting sigmoidal curve we calculated the peptide IC_50_ to be equal to 58 µM (Figure 3f).

### 3.3 Determination of peptide binding affinity by SPR

**FIGURE 4:**
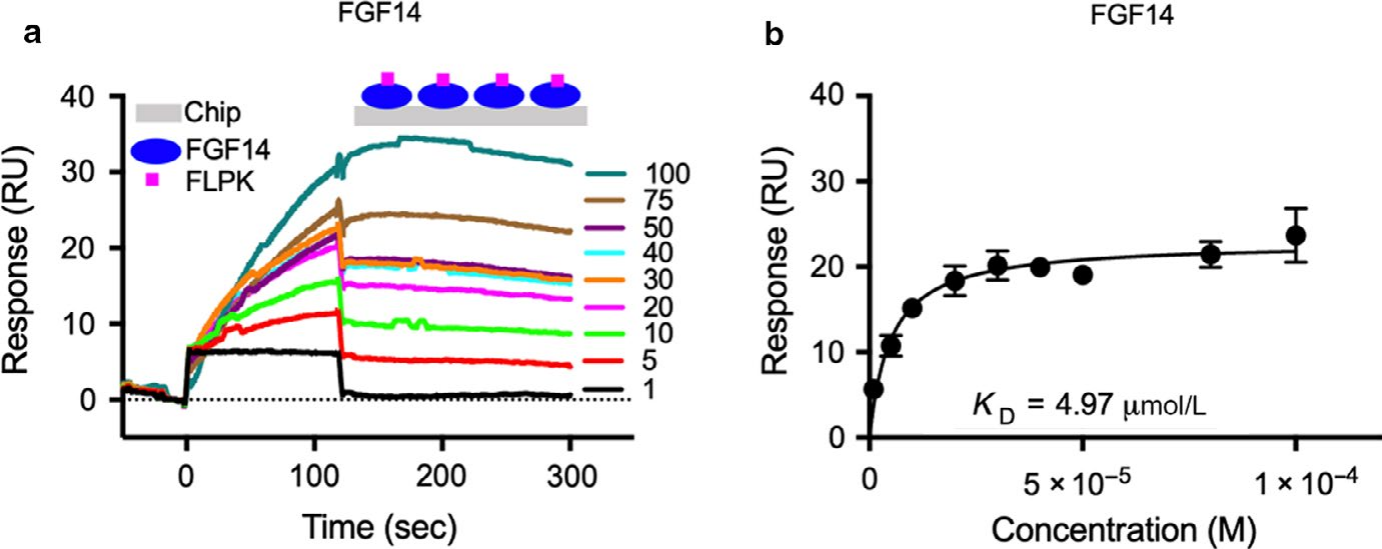
FLPK binding to FGF14 assessed by surface plasmon resonance. (a) representative SPR sensorgram of FLPK (1–100 µM) binding to FGF14. (b) average saturated binding curve for FLPK binding to FGF14 for three independent experiments. FGF14 purified protein (RU 16 000) was immobilized (using the Amine Coupling Kit, see Methods) on CM5 sensor chips, and FLPK was flown over the chip using a flow rate of 50 µl/min. Data are mean ± *SEM* in panels (b)

The LCA revealed that all three tetrapeptides are capable of interfering with the FGF14:Nav1.6 complex formation, and that FLPK and EYYV likely do so via interaction with V160. Thus, we proceeded to evaluate binding of peptides to *E*. *coli* purified FGF14, FGF14^V160A^ and Nav1.6 C‐terminal tail proteins using SPR. FGF14, FGF14^V160A^ or the Nav1.6 C‐terminal tail were coupled with biosensor chip CM5 and increasing concentrations of peptides were flew over the chip (Figure 4a). We found that FLPK binds to FGF14 in low micro‐molar range with an estimated affinity of K_D_ = 4.97 µM, calculated from resonance unit (RU) values at the steady‐state (Figure 4b). We observed very high K_on_ (84.41 ± 0.94 M^−1^ s^−1^) and low K_off_ [(4.31 ± 4.4) × 10^–4^] for FLPK concentrations (1–100 µM) suggesting high affinity of peptide with FGF14. In contrast, FLPK showed no measurable binding to either the Nav1.6 C‐terminal tail or FGF14^V160A^ (Table 2), which indicates very low affinity of FLPK to these proteins. The other two peptides, PLEV and EYYV, were also tested for direct binding to FGF14 or Nav1.6 C‐tail, and no appreciable affinity to either protein was detected (Table 2). These data suggest that FLPK regulates formation of the FGF14:Nav1.6 complex by interacting with V160, a key hotspot at the protein:channel interface as reported previously (Ali et al., 2016).

**TABLE 2:** Kinetic parameters for the binding of peptides to FGF14 or Nav1.6 C‐terminal tail

Protein–Peptide Interaction	k_on_ (M^−1^s^−1^)	k_off_ (s^−1^)	K_D_ (M)
FLPK‐FGF14	(84.41 ± 0.94)	(4.31 ± 4.4) X 10^–4^	(4.97 ± 0.64) X 10^–6^
PLEV‐FGF14	N.M.	N.M.	—
EYYV‐FGF14	N.M.	N.M.	—
FLPK‐FGF14^V160A^	N.M.	N.M.	—
FLPK‐Nav1.6	N.M.	N.M.	—
PLEV‐Nav1.6	N.M.	N.M.	—
EYYV‐Nav1.6	N.M.	N.M.	—

Abbreviations: K_D_, equilibrium constant;k_off_, dissociation constant; k_on_, association constant.

N.M., not measurable; (—), not determinable.

### 3.4 FLPK peptide modulates Nav1.6 channel activity in the presence of FGF14

**FIGURE 5:**
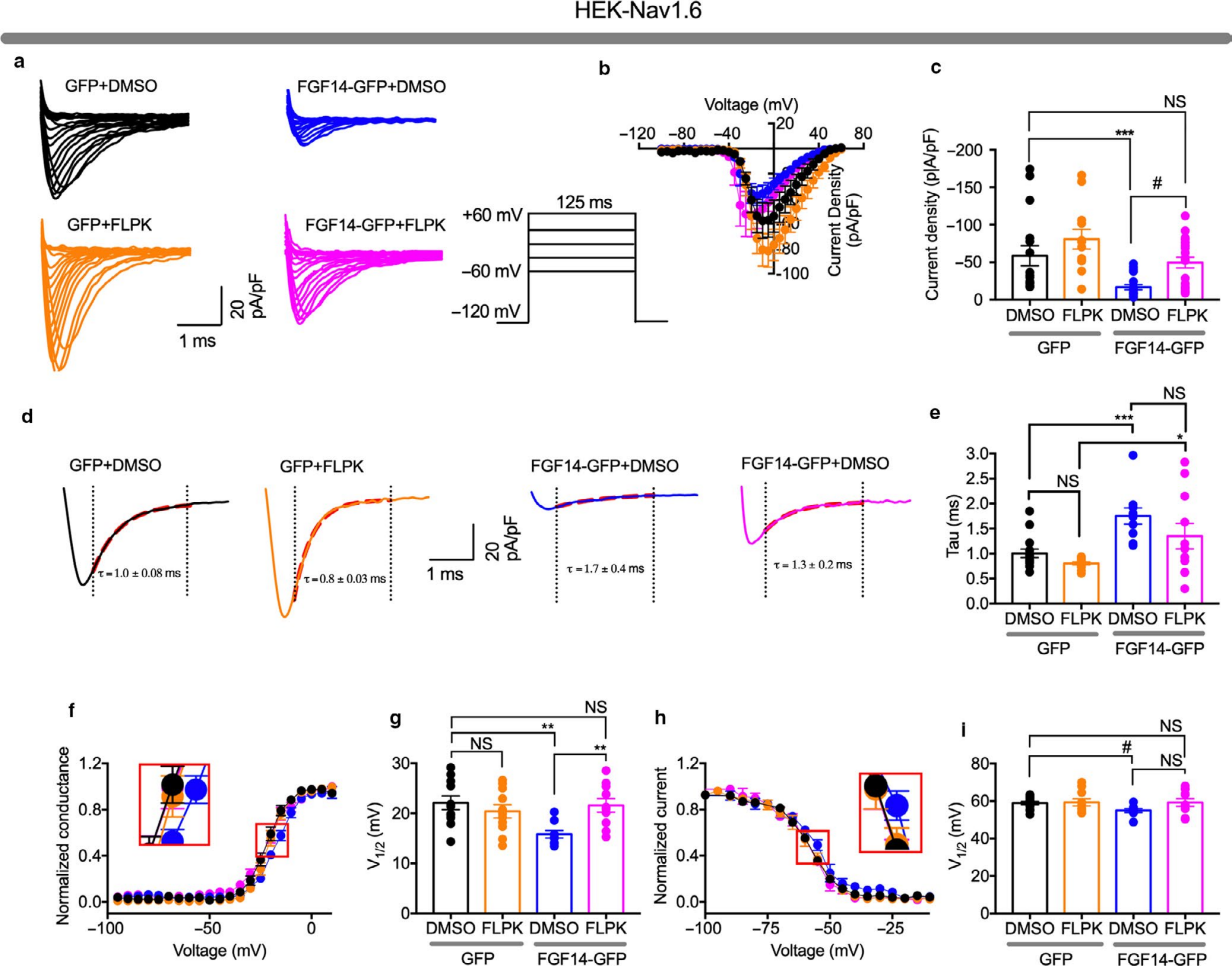
FLPK inhibits FGF14‐dependent modulation of Nav1.6 currents. (a) representative traces of voltage‐gated Na^+^ currents (*I*_Na_) recorded from HEK‐Nav1.6 cells transiently expressing GFP or FGF14‐GFP in response to voltage steps from −120 mV to + 60 mV from a holding potential of −70 mV (*inset*). GFP‐expressing cells were treated with DMSO (*black*) or with 50 μM FLPK (*orange*), whereas FGF14‐GFP‐expressing cells were treated either with DMSO (*blue*) or with 50 μM FLPK (*magenta*). (b) current–voltage relationships of *I*_Na_ from the experimental groups described in (a). (c) bar graphs representing peak current densities measured at −10 mV in cells expressing GFP treated with DMSO (*black*) or 50 μM FLPK (*orange*) or expressing FGF14‐GFP treated with DMSO (*blue*) or 50 μM FLPK (*magenta*). (d) representative traces from experimental groups described in (a) in which tau (τ) of *I*_Na_ was measured using a one‐term exponential fitting function (red dotted line). (e) summary bar graph of τ calculated at peak currents at −10 mV in the indicated experimental groups. (f) voltage‐dependence of activation is plotted as a function of the membrane potential (mV); FGF14‐GFP‐expressing cells were treated either with DMSO (*blue*) or 50 μM FLPK (*magenta*). Data were fitted with the Boltzmann equation as indicated in the experimental section. (g) summary bar graph of V_1/2_ of activation in the indicated experimental groups. (h) voltage‐dependence of steady‐state inactivation is measured using a two‐step protocol and relative current plotted as a function of the membrane potential (mV); data were fitted with the Boltzmann equation as indicated in the experimental section. (i) summary bar graph summary of V_1/2_ of steady‐state inactivation in the indicated experimental groups. The fitted parameters are provided in Table 3. Data are mean ± *SEM*. *p < .05, **p < .001; ^#^ *p* < .005; NS = nonsignificant. Student's *t* test, one‐way ANOVA post hoc Dunn test and post hoc Fisher's LSD

To test whether FLKP had any modulatory effects on Nav1.6‐mediated Na^+^ currents, we used whole‐cell patch‐clamp electrophysiology in HEK293 cells stably expressing Nav1.6 (HEK‐Nav1.6) that were transiently transfected with either *GFP* (HEK‐Nav1.6 GFP) or *FGF14‐1b‐GFP* (HEK‐Nav1.6 FGF14‐GFP); each group was either treated with FLPK (50 μM from a stock solution dissolved in DMSO) or 0.1% DMSO alone (vehicle) (Figure 5a‐c). In GFP cells treated with FLPK, Nav1.6‐mediated transient Na^+^ currents (*I*_Na_) were not statistically different (−80.8 ± 12.2 pA/pF, *n = *14) compared to vehicle (−58.8 ± 13.6 pA/pF*, n* = 16, *p* < .1330; Figure 5a‐c). However, in the FGF14‐GFP group, in which, as expected (Ali et al., 2016, 2018; Shavkunov et al., 2013), *I*_Na_ were strongly suppressed compared to control (−16.8 ± 3.3 pA/pF, *n = *19, *p* < .001, One‐Way ANOVA, post hoc Dunn's test), FLPK effectively restored *I*_Na_ to values that were statistically comparable to the GFP + DMSO control (FLPK: −49.6 ± 7.2 pA/pF, *n = *21; *p* = .5294), as illustrated in Figure 5a‐c. The decay time constant (τ) of *I*_Na_ in the GFP group was minimally affected by FLPK (0.8 ± 0.03 ms, *n* = 12) compared to vehicle (1.0 ± 0.08 ms, *n* = 15; *p* = .0495), but was significantly slower in the presence of FGF14‐GFP (1.7 ± 0.4 ms, *n* = 10*; p* < .05 compared to the GFP control group) and restored to the DMSO GFP control group by FLPK (1.3 ± 0.2 ms, *n* = 11; *p* < .05; Figure 5d,e and Table 3). As previously described (Ali et al., 2016, 2018; Shavkunov et al., 2013), we also observed a depolarizing shift in the V_1/2_ of Nav1.6 activation and steady‐state inactivation induced by FGF14, phenotypes that were both prevented by FLPK (V_1/2_ activation: −16.6 ± 1.0 mV, *n* = 10 for vehicle versus. −20.8 ± 1.5 mV, *n* = 11; *p* < .001 for FLPK; Figure 5f,g; V_1/2_ steady‐state inactivation: −58.9 ± 0.8 mV, *n* = 14; Figure 5h‐i).

**TABLE 3:** Effect of FLPK on Nav1.6‐mediated currents in the presence of FGF14 or ΔNT‐FGF14

Condition	Peak density	Activation	Kact	Steady‐state Inactivation	Kinact	Tau (τ)
	pA/pF	mV	mV	mV	mV	ms
GFP (DMSO)	−58.6 ± 13.9 (15)	−21.8 ± 1.3 (12)	4.3 ± 0.2 (12)	−58.9 ± 0.8 (14)	5.8 ± 0.4 (14)	1.0 ± 0.08 (15)
GFP (FLPK)	−80.8 ± 12.2 (14)	−20.5 ± 1.2 (12)	3.9 ± 0.3 (12)	−59.3 ± 1.9 (10)	6.1 ± 0.5 (10)	0.8 ± 0.03 (12)r
FGF14‐GFP (DMSO)	−16.8 ± 3.3 (19)^a,b^	−16.6 ± 1.0 (10)^d^, ^e^	5.7 ± 0.4 (10)^f,g^	−54.9 ± 1.1 (8)^j^	5.9 ± 0.9 (8)	1.7 ± 0.4 (10)^k^
FGF14‐GFP (FLPK)	−49.6 ± 7.2 (21)^c^	−20.8 ± 1.5 (11)	5.7 ± 0.5 (11)^h,i^	−59.2 ± 2.1 (11)	5.9 ± 0.6 (14)	1.3 ± 0.2 (11)
ΔNT‐FGF14‐GFP (DMSO)	–108 ± 18.4 (17)^n^	–21.2 ± 0.36 (14)	4.61 ± 0.3 (14)	–70.2 ± 0.53 (13)^o,p,q^	6.4 ± 0.44 (13)	1.091 ± 0.07 (15)
ΔNT‐FGF14‐GFP (FLPK)	−126.04 ± 12.3 (11)^m^, ^NS^	–37.43 ± 0.3 (11)^l^, ^o,p,q^	1.63 ± 0.23 (11)	–68.3 ± 0.34 (11)^o,p,q^	6.75 ± 0.32 (11)	1.137 ± 0.08 (11)

Note

Data are mean ± *SEM*.

^a,b^ *p* < .001, one‐way ANOVA, post hoc Dunn test compared with GFP (DMSO) and (FLPK).

^c^ *p* < .05, unpaired *t* tests compared to FGF14‐GFP (DMSO).

^d^ *p* < .01, one‐way ANOVA, post hoc Fisher's LSD compared with GFP (DMSO).

^e^ *p* < .05, one‐way ANOVA, post hoc Fisher's LSD compared with GFP (FLPK).

^f,g^ *p* < .01, one‐way ANOVA, post hoc Fisher's LSD compared with GFP (DMSO) and (FLPK).

^h,i^ *p* < .01, one‐way ANOVA, post hoc Fisher's LSD compared with GFP (DMSO) and (FLPK).

^j^ *p* < .05, unpaired *t* tests compared to GFP (DMSO).

^k^ *p* < .05, one‐way ANOVA, post hoc Dunn test compared with GFP (DMSO).

^l^ *p* < .001, unpaired *t* tests compared to FGF14‐ΔNT‐GFP (DMSO).

^m^ *p* < .001, unpaired *t* tests compared to Nav1.6‐GFP (DMSO).

^n^ *p* < .05, unpaired *t* tests compared to Nav1.6‐GFP (DMSO).

^o,p,q^ *p* < .001, unpaired *t* tests compared to Nav1.6‐GFP (DMSO).

^r^ *p* = .0495, unpaired *t* tests compared to Nav1.6‐GFP (DMSO).

^NS^ *p* < .5, unpaired *t* tests compared to FGF14‐ΔNT‐GFP (DMSO).

### 3.5 The modulatory activity of Nav1.6 currents by FLPK requires the FGF14 N‐terminal domain

**FIGURE 6:**
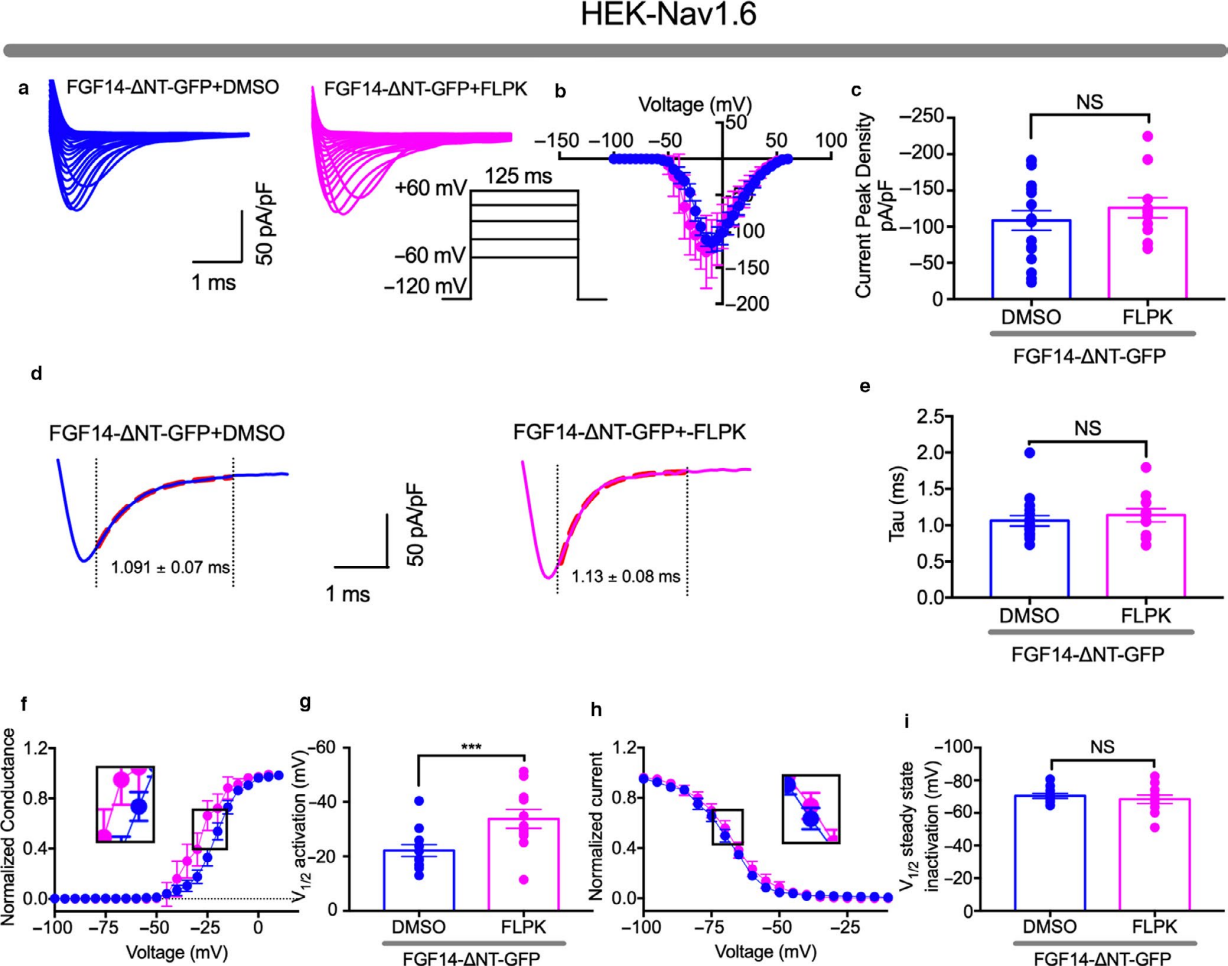
FLPK functional activity depends on the FGF14 N‐terminal tail. (a) representative traces of voltage‐gated Na^+^ currents (*I*_Na_) recorded from HEK‐Nav1.6 cells transiently expressing FGF14‐ΔNT‐GFP in response to voltage steps from −120 mV to +60 mV from a holding potential of −70 mV (*inset*). FGF14‐ΔNT‐GFP‐expressing cells were treated with either DMSO (*blue*) or 50 μM FLPK (*magenta*). (b) current‐voltage relationships from the experimental groups described in (a). (c) summary bar graphs representing peak current densities measured at −10 mV in cells expressing FGF14‐ΔNT‐GFP treated with either DMSO (*blue*) or 50 μM FLPK (*magenta*). (d) representative traces of experimental groups described in panel A in which tau (τ) of *I*_Na_ was measured using a one‐term exponential fitting function (red dotted line). (e) summary bar graph of τ calculated at peak currents at −10 mV in the indicated experimental groups. (f) voltage‐dependence of activation is plotted as a function of the membrane potential (mV); FGF14‐ΔNT‐GFP‐expressing cells were treated either with DMSO (*blue*) or 50 μM FLPK (*magenta*). Data were fitted with the Boltzmann function as indicated in the experimental section. (g) bar graph summary of V_1/2_ of activation in the indicated experimental groups. (h) steady‐state inactivation is measured using a two‐step protocol and relative current plotted as a function of the membrane potential (mV); data were fitted with the Boltzmann function as indicated in the experimental section. (i) summary bar graph summary of V_1/2_ of steady‐state inactivation in the indicated experimental groups. The fitted parameters are provided in Table 3. Data are mean ± *SEM*. NS = nonsignificant. ***p < .001. Student's *t* test

The FGF14‐1b N‐terminal domain plays a crucial role in regulating Nav1.6 currents (Ali et al., 2018). Thus, we sought to determine whether this domain effects the activity of FLPK. We tested this hypothesis using the same experimental design of Figure 5 except that HEK‐Nav1.6 cells were transiently transfected with an FGF14 lacking the N‐terminal domain (FGF14‐ΔNT‐GFP). These cells exhibited *I*_Na_^+^ that were larger compared to FGF14‐GFP (Figure 6a, Table 3) and insensitive to FLPK (−121.9 ± 12.5 pA/pF, *p* < .40, *n* = 12; Figure 6a‐c). Except for inducing a hyperpolarizing shift of V_1/2_ activation (–11.6 mV, *p* < .0006, *n* = 11 and *n* = 12, Figure 6d,e), FLPK had no effects on any other parameters associated with FGF14‐GFP regulation such as V_1/2_ of steady‐state inactivation (Figure 6g,h) or τ of fast inactivation (Figure 6d,e). Detailed analysis of data presented in Figure 6 is summarized in Tables 3 and 4. Overall, these results suggest that despite binding to the FGF14 core domain (Figure 4, Table 2), FLPK functional activity requires the FGF14 N‐terminal tail.

**TABLE 4:** Effect of FLPK on MSNs intrinsic excitability and passive electrical properties

Condition	RMP	I_thr_	V_thr_	Cm	R_in_	Tau (τ)
	mV	pA	mV	pF	MΩ	ms
DMSO	−75.3 ± 1.5	110 ± 14.2	−40.5 ± 1.1	90.2 ± 14.5	119.1 ± 10.3	16.1 ± 2.1
FLPK	−79.7 ± 2.6	78 ± 18.3	−38.7 ± 2.2	80.1 ± 7.6	147.2 ± 11.3	11.9 ± 1.6
*p* value	.125^a^	.221^a^	.422^a^	.521^a^	.088^a^	.157^a^

^a^ Two‐tailed *t* test; data are mean ± *SEM*; *n* = 5–12.

### 3.6 Effect of FLPK on intrinsic excitability of MSNs

**FIGURE 7:**
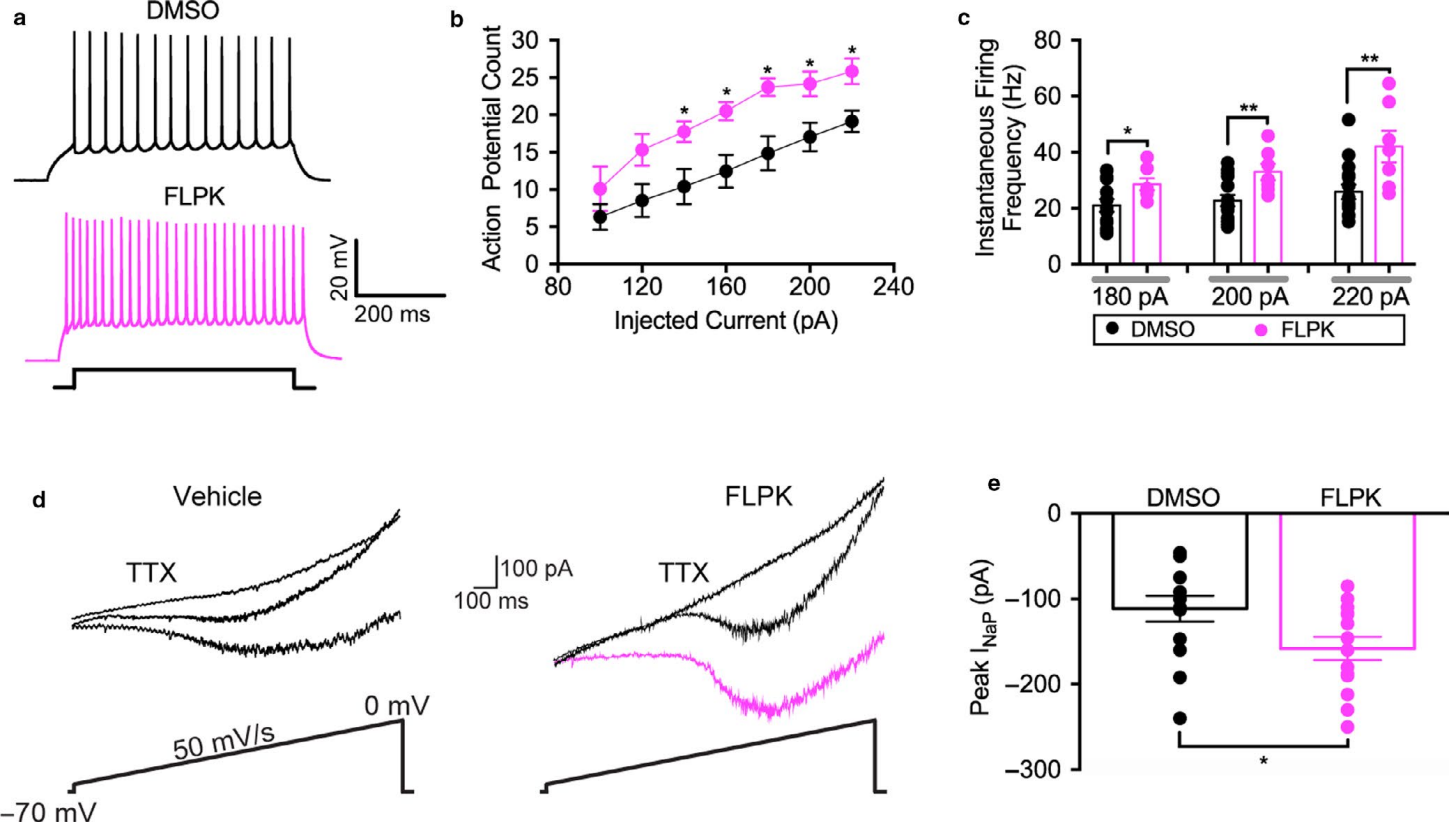
FLPK modulates intrinsic excitability and persistent sodium current of MSNs in the NAc. (a) representative traces of action potentials (AP) evoked at a current step of 180 pA in MSNs treated with either 0.1% DMSO (*black*) or 50 μM FLPK (*magenta*). (b) average action potential count at varying injected current stimuli recorded in MSN in response to 0.1% DMSO (*black*) or 50 μM FLPK (*magenta*). (c) average instantaneous firing frequency (IFF) at varying injected current stimuli recorded in MSN in response to 0.1% DMSO (*black*) or 50 μM FLPK (*magenta*). (d) rrepresentative traces of MSN *I*_NaP_ elicited by application of slow voltage ramps (50 mV/s) in the presence (*right*) or absence (*left*) of FLPK (50 µM). *I*_NaP_ was isolated by digital subtraction of responses obtained in the presence of 0.5 μM TTX from those recorded under control conditions. (e) summary bar graph showed the experimental groups in d). Data are mean ± *SEM* (*n* = 7–14); *p < .05, ***p < .001; Student's *t* test and Mann–Whitney Test

Finally, we investigated the effect of FLPK on intrinsic excitability of medium spiny neurons (MSNs) in the nucleus accumbens (NAc). Previous work in our laboratory has shown both FGF14 and Nav1.6 to be expressed and colocalized within the axon initial segment of MSNs, the subcellular determinant of neuronal excitability (Ali et al., 2018). We utilized whole‐cell patch‐clamp electrophysiology to measure intrinsic firing properties of MSNs following acute incubation with 50 µM FLPK compared to vehicle (DMSO 0.1%). FLPK significantly increased the number of evoked action potentials and the instantaneous firing frequency in MSNs across a series of injected currents (Figure 7a‐c). At current step 180 pA, the number of action potentials was 23.7 ± 1.2, *n* = 7 for the FLPK group versus 14.9 ± 2.3, *p* < .016, *n* = 14 for DMSO (Figure 7b). At current step 180 pA, the instantaneous firing frequency was 28.6 ± 2.1 Hz, *n* = 7 for FLPK versus 19.7 ± 2.6 Hz, *p* < .0395, *n* = 14 for DMSO (Figure 7c). We also identified an increase in persistent sodium current (*I*_NaP_) from MSNs treated with FLPK (−158.2 ± 14.2 mV versus control 111.4 ± 15.7 pA, *p* < .03, *n* = 14, Figure 7d,e). As shown in Table 5, there were no changes in several other electrophysiological parameters including membrane potential and input resistance, indicating that the effects of FLPK on MSN firing can be attributed to the observed potentiation of *I*_NaP_.

## 4 DISCUSSION

Mapping PPI interfaces within macromolecular complexes using chemical probes is an emerging approach to study ion channels (Ali et al., 2016; Ma & Nussinov, 2014; Nanaware, Ramteke, Somavarapu, & Venkatraman, 2014; Petit et al., 2018) that can lead to drug discovery campaigns with potential therapeutic value (Miller et al., 2017; Petit et al., 2018; Wadsworth et al., 2019). Along these lines, we used in silico docking, LCA, SPR and whole‐cell patch‐clamp electrophysiology in heterologous cells and in the intact brain circuit to design, synthetize and characterize the activity of a tetrapeptide mapped to the FGF14:Nav1.6 PPI interface. These studies provide insights into the regulatory mechanisms of the Nav1.6 channel by a relevant accessory protein, FGF14, which could have implications for diagnostic and therapeutic development against channelopathies associated with Nav1.6 and FGF14 (Di Re et al., 2017; Hoxha, Balbo, Miniaci, & Tempia, 2018; Meisler, Kearney, Escayg, Macdonald, & Sprunger, 2001; O'Brien & Meisler, 2013).

### 4.1 In silico docking and in‐cell assays reveal binding of FLPK to FGF14

Using in silico docking, in‐cell LCA and SPR, combined with model‐guided site‐directed mutagenesis, we evaluated the activity of three short peptides mapped to the PPI interface of the FGF14:Nav1.6 channel complex. In silico studies of the tetrapeptides FLPK, PLEV and EYYV predicted interactions with previously characterized hotspots at the PPI interface of the FGF14:Nav1.6 complex. FLPK docked to the β9 sheet within the inner pocket of FGF14 and on the β12 sheet (P203) (Figure 1 and Table 1), whereas PLEV and EYYV interacted primarily with residues on the β5 sheet and N‐terminus of FGF14 (Ali et al., 2016, 2018; Chang et al., 2006; Goetz et al., 2009; Wang, Chung, Yan, Lee, & Pitt, 2012). Specifically, FLPK docked to the inner pocket through binding of Lys to N157 via H‐bonding, Y158 via both H‐bonding and π‐cation effects (Figure 1c,e), as well as V160 via hydrophobic interactions (Figures 1 and 2c). While EYYV and PLEV demonstrated minimal binding to the inner pocket (no interactions with Y158 or Y159), they were predicted to bind more peripheral domains of FGF14 (β5 sheet and N‐terminus) that are also key sites of interaction with Nav1.6 (Ali et al., 2016).

When tested in vitro using LCA, all three peptides exhibited a comparable degree of inhibition of the FGF14:Nav1.6 complex assembly (Figure 3a,b). However, only the activity of FLPK and EYYV, and not PLEV, was abolished by introducing single FGF14^V160A^ or double FGF14^Y158A/V160A^ mutations (Figure 3c‐e). These LCA results were consistent with docking poses that predict binding of FLPK to Y158 and V160, two previously identified hotspots at the FGF14:Nav1.6 complex interface (Figure 3c,d). Furthermore, the effect of FLPK is similarly abolished when replacing these residues with asparagine (FGF14^Y158N/V160N^), but not for PLEV or EYYV (Figure 3e). This indicates that binding of FLPK requires both hydrophobic and electrostatic interactions, abolished in the FGF14^Y158N/V160N^ and FGF14^Y158A/V160A^ double mutant respectively, a finding in support of our docking (Figure 1e). Binding of FLPK to V160 could occur through stabilization of the phenyl ring (of FLPK) via π‐cation interactions with Y158, as well as lysine‐mediated interactions including H‐bonding, a mechanism shared by other Nav channel regulators (Cahalan & Almers, 1979; Yeh & Narahashi, 1977; Zamponi & French, 1994).

Alongside LCA data, these findings indicate that the hotspots Y158 and V160 play a crucial role in stabilizing the complex (Ali et al., 2016; Jiang & Lai, 2002; Ochiai et al., 2011; Wang et al., 2012) and establish FLPK as a specific probe targeting these residues. LCA studies also revealed more complex protein–peptide interactions such as for PLEV with the FGF14^Y158A^:Nav1.6 channel complex. This could be the result of either competition between Y158 and PLEV for binding to Nav1.6 or direct binding of the peptide to the mutated site (Y158A) (Figure 3c‐e). Further studies are required to provide a more accurate model of PLEV mechanism of action. Additionally, dose‐response studies with FLPK showed that the tetrapetide is able to reduce the FGF14:Nav1.6 complex formation with an apparent IC_50_ = 58 μM, but a relatively small efficacy of above 50% compared to control conditions (Figure 3f), indicative of weak inhibition. To further understand the protein:peptide interactions, we employed SPR as an orthogonal approach to determine binding affinity of the peptides to either FGF14 or the Nav1.6 C‐terminal tail in vitro. Of the three tested peptides, only FLPK exhibited measurable affinity (K_D_ = 4.97 μM) for FGF14 (Figure 4a,b), and none of the peptides bound to the Nav1.6 C‐terminal tail. Furthermore, no binding of FLPK was detected for the FGF14^V160A^ mutant (Table 2). These results confirm V160 as a necessary binding site of FLPK, while also indicating that the peptide requires minimal structural surface area for binding, an attribute that has been shown to correlate with a high degree of target specificity (Chang et al., 2006; Ling, Liao, Clark, Wong, & Lo, 2008; Spiga et al., 2002).

### 4.2 Functional activity of FLPK in heterologous cells expressing the Nav1.6 channel

Whole‐cell patch‐clamp electrophysiology was employed to evaluate the effect of FLPK on phenotypes of Nav1.6 currents induced by FGF14. While FLPK had no measurable effects in cells expressing Nav1.6 alone (HEK‐Nav1.6 GFP), it inhibited previously reported modulatory effects of FGF14 (1b isoform) on Nav1.6 currents (Figure 5a‐c,h,i; Tables 3,4), including suppression of peak transient currents, decrease in the rate of fast inactivation, and depolarizing shifts in voltage‐dependence of steady‐state inactivation (Ali et al., 2016, 2018). In the absence of the FGF14‐1b N‐terminal domain peak transient currents, rate of fast inactivation, and voltage‐dependence of steady‐state inactivation were unaffected by FLPK (Figure 6a‐e,h,i) suggesting that despite binding to the FGF14 core domain, FLPK requires the N‐terminal tail of FGF14 to exert its activity.

The phenotype induced by FLPK on V_1/2_ of activation is more complex. While in both FGF14 wild type and FGF14‐ΔNT cells, FLPK induced a shift of V_1/2_ of activation in the hyperpolarizing direction (−4 mV and −16 mV, respectively, compared to the corresponding DMSO control group, Table 3), the magnitude of the phenotype was significantly greater in FGF14‐ΔNT cells. A mechanism to account for these differences could be a competition between FLPK and the FGF14 N‐terminal tail in regulating Nav1.6 activation with two opposing mechanisms: hyperpolarizing shift induced by FLPK, depolarizing shift driven by the N‐terminal tail. In cells expressing the FGF14 wild type protein, the mechanism regulating voltage‐dependence of activation might simply result from a balance of these two mechanisms, while in FGF14‐ΔNT cells it might be solely driven by FLPK resulting in a greater hyperpolarizing shift. Alternatively, in the absence of the FGF14 N‐terminal tail FLPK might gain access to amino acid residues normally buried resulting in an effect on channel activation that would be normally precluded in the presence of the wild type protein. Taken together, these findings clearly indicate a dependency of FLPK functional activity on the FGF14 N‐terminal tail. However, it remains to be determined how these peptide:protein interactions impact the aforementioned phenotypes on Nav1.6 and to which extent they translate to the native system model where complexity is greater than heterologous cells.

### 4.3 Role of FLPK in regulating Na^+^ currents and excitability in MSNs

Extensive evidence has been provided for a role of FGF14‐1b in suppressing Na^+^ current in heterologous cells, such as HEK293 cells (Ali et al., 2016, 2018; Laezza et al., 2009; Lou et al., 2005; Shavkunov et al., 2013). This phenotype, unique to the FGF14‐1b splice variant, is opposite to the postulated role of FGF14 in the native system which is to promote Nav channel activity. These seemingly opposite phenotypes can be reconciled by considering cell background differences, variations in the FGF14‐1b protein conformation or the presence of other accessory proteins that could make up for the complex role FGF14 may have in the native system. Thus, while studies in HEK293 cell have been pivotal in parsing out key structural regulatory elements of the FGF14:Nav1.6 PPI interface, experimental findings in heterologous cells should be interpreted cautiously. Despite these limitations, the substantial ability of FLPK to modulate Nav1.6 in heterologous cells provided a strong premise to evaluate the effect of the tetrapetide in neurons.

We choose to study the effect of FLPK in MSNs in the NAc, where both FGF14 and Nav1.6 are abundantly expressed and required for intrinsic firing (Ali et al., 2018; Scala et al., 2018). Treatment with FLPK led to an increase in the total number of action potentials as well as instantaneous firing frequency evoked in response to current steps (Figure 7a‐c; Table 4). In addition, FLPK potentiated persistent Na^+^ currents (Figure 7d,e; Table 4). Taken together with the lack of observed effect on R_input_ and resting membrane potential, this phenotype provides direct evidence for potentiation of persistent Na^+^ currents as a plausible mechanism for FLPK in controlling MSN firing.

Previous studies have shown that overexpression of FGF14‐1b in hippocampal neurons potentiates transient Na^+^ currents (Laezza et al., 2007), while genetic deletion of FGF14 leads to reduced Na^+^ currents and firing in several neuron subtypes including MSNs in the NAc (Ali et al., 2018; Goldfarb et al., 2007; White et al., 2019). These phenotypes have been attributed to a role of FGF14 in preserving the pool of active channels, in part by minimizing inactivation and increasing channel availability (White et al., 2019). However, if FLPK acts as an inhibitor of the FGF14:Nav1.6 complex, as suggested by our LCA studies, the effect of the tetrapeptide on persistent Na^+^ currents would be hard to reconcile. A plausible hypothesis to explain its mechanism of action in the native system is that rather than limiting the FGF14:Nav1.6 complex formation, which would result in suppression of Na^+^ currents and decreased firing, FLPK minimizes channel inactivation by stabilizing or altering interactions of FGF14 with other domains of Nav1.6 independently from the core domain like the N‐terminal tail, which is supported by our studies in heterologous cells.

Evidence has been provided for roles of the N‐terminal tail of the iFGFs‐1a splice variants (which diverge from the 1b isoforms only at the N‐terminal tail) on Nav channel inactivation independently of their core domain (Dover, Solinas, D'Angelo, & Goldfarb, 2010; Venkatesan, Liu, & Goldfarb, 2014; White et al., 2019). However, the N‐terminal tail of the FGF14‐1b splice variant bears no sequence homology with any other members of the iFGFs family. Thus, the phenotypes observed here induced by FLPK might not be directly comparable to the ones reported previously and could represent a novel mechanism of regulation of Nav channels by FGF14‐1b which needs to be further explored. FLPK could also potentially bind to the core domain of the FGF14‐1a splice variant, such that the phenotype reported here would reflect an effect of the tetrapeptide on FGF14‐1a. However, FGF14‐1b is the predominant splice variant expressed in the adult brain, and as such is likely the most relevant protein product in MSNs (Munoz‐Sanjuan, Smallwood, & Nathans, 2000; Wang, McEwen, & Ornitz, 2000; Yamamoto, Tadahisa, Norihiko, Mitsuhiro, & Nobuyuki, 1998).

The presence of other accessory proteins that directly interact with Nav1.6 in the native system may contribute to the reported activity of FLPK. A critical determinant required for repetitive firing in MSN and Purkinje neurons is the Navβ4 accessory subunit (Ji et al., 2017; White et al., 2019). Peptide sequences derived from the LITFILKK motif of the cytoplasmic tail of Navβ4 prevent stable fast inactivation and generate resurgent and persistent Na^+^ currents through a voltage‐dependent open‐channel block, facilitating repetitive firing (Grieco, Malhotra, Chen, Isom, & Raman, 2005; White et al., 2019). Structurally, FLPK shares similarities with the LITFILKK motif. In both, the aromatic phenylalanine is adjacent to hydrophobic residues (leucine and isoleucine) and is equidistant from the basic lysine residues. Combined, this could mimic the chemical signature of β4 such that FLPK could substitute for the LITFILKK motif minimizing fast inactivation and promoting Na^+^ persistent currents and repetitive firing in MSNs.

## 5 CONCLUSIONS

We have studied the properties of FLPK, a tetrapeptide derived from the FGF14 sequence at a site that mediates PPI of the core domain of FGF14 with the Nav1.6 C‐terminal tail. Our studies suggest that FLPK binds hotspot residues on the FGF14 core domain, which were previously shown to modulate Nav1.6 function, but that its primary mechanism of action depends on the FGF14 N‐terminal tail. By characterizing FLPK activity in the native system we discovered that FLPK increased MSN firing and potentiated persistent Na^+^ currents supporting a model by which, rather than limiting the FGF14 core domain interaction with the Nav channel FLPK inhibits Nav channel inactivation. Future studies will use FLPK to probe for roles of the FGF14‐1b in regulating Nav channel inactivation and repetitive firing.

## CONFLICT OF INTEREST

Dr. Fernanda Laezza is a founder and owns stocks in IonTx Inc.

## AUTHOR CONTRIBUTIONS

AKS and FL designed the experiments. AKS performed the mutagenesis, protein purification and SPR. AKS and PAW performed the LCA. AKS, and SRA performed patch‐clamp electrophysiology. AKS and CT performed acute brain slice electrophysiology. HC and PAW performed the homology modeling and docking. AKS, PAW, CT, and FL wrote the manuscript. All authors contributed to edit the manuscript.
